# Survival in advanced GIST has improved over time and correlates with increased access to post-imatinib tyrosine kinase inhibitors: results from Life Raft Group Registry

**DOI:** 10.1186/s13569-019-0114-5

**Published:** 2019-04-02

**Authors:** Jerry W. Call, Yu Wang, Denisse Montoya, Norman J. Scherzer, Michael C. Heinrich

**Affiliations:** 1 Life Raft Group, 155 Route 46 West, Suite 202, Wayne, NJ 07470 USA; 2 Portland VA Health Care System and Knight Cancer Institute, Oregon Health & Science University, Portland, OR USA

**Keywords:** Gastrointestinal stromal tumors, GIST, Regorafenib, Sunitinib, Survival

## Abstract

**Background:**

The use of imatinib, sunitinib, and regorafenib has transformed the treatment of advanced GIST. Sunitinib and regorafenib improve progression free-survival in the second (2L) and third (3L) line, respectively, compared with placebo. However, the impact of these agents on overall survival (OS) is unclear.

**Methods:**

The Life Raft Group (LRG) patient registry contains records from 1716 GIST patients; 526 have advanced to at least 2L treatment. Patient-reported treatment and outcome data were examined to determine treatment patterns and their impact on OS.

**Results:**

Median OS from start of 2L therapy was 32.4 months for sunitinib (n = 436) compared with 27.1 months for patients treated with any other 2L drug (n = 74, *p* = 0.023, HR 1.377) and 16.8 months for patients who never received sunitinib in any treatment line (n = 42, *p* = 0.028, HR 1.52). In patients reporting progression in 2L, the median OS in patients subsequently receiving 3L regorafenib (n = 53, 26.2 months) was longer than that of 3L patients who never received regorafenib in any line of therapy (n = 174, 14.3 months, *p* = 0.0002, HR 2.231), and was longer than that of patients who received any other 3L treatment (19.8 months, *p* = 0.044, HR 1.525). OS for advanced GIST patients in the LRG registry has improved over time (*p* = 0.0013), correlated with the increased use of TKIs in ≥ 2L settings.

**Conclusions:**

In our analysis, sunitinib and regorafenib significantly improved OS compared with patients who never received these agents. Our data also support the hypothesis that the use of KIT/PDGFRA inhibitors, including non-approved agents, has improved OS for patients with imatinib- and sunitinib-resistant GIST.

**Electronic supplementary material:**

The online version of this article (10.1186/s13569-019-0114-5) contains supplementary material, which is available to authorized users.

## Background

Gastrointestinal stromal tumors are soft tissue sarcomas that arise from interstitial cells of Cajal (ICC) or from stem cells that can differentiate towards ICCs. GIST have a reported incidence of approximately 14.5 per million per year [1]. Primary tumors most commonly originate in the stomach or intestines and frequently metastasize to the liver or the peritoneum [2, 3].

Following successful clinical studies of imatinib which began in 2000 [4, 5], treatment with tyrosine kinase inhibitors (TKIs) became the standard treatment for advanced GIST. TKI’s currently approved for GIST in the United States and many other countries include imatinib [6], sunitinib [7] and regorafenib [8]. Imatinib is approved for first line (1L) therapy for advanced/metastatic GIST and for adjuvant treatment after surgery. Sunitinib is approved after progression on or intolerance to imatinib [7]. Regorafenib is approved for patients previously treated with imatinib and sunitinib [8, 9].

Approximately 75 to 80% of GISTs have activating mutations in *KIT* and another 5–8% harbor mutations in *PDGFRA* [10]. These mutations are felt to be the pathogenic event that initiates tumor formation [11]. TKIs that inhibit the signaling from these mutated proteins are the primary drug treatment for GISTs. The most common cause of GIST resistance to imatinib, and other TKIs, is secondary mutations in the driver mutant kinase [12]. In *KIT*, these secondary mutations most commonly occur in exons 13, 14, 17 and 18 [12, 13]. The ability of sunitinib and regorafenib to inhibit, at least some, of these secondary mutations provides the likely basis for their antitumor activity in imatinib-resistant GIST patients [13, 14].

The reported survival times for GIST patients vary widely depending on stage of disease [15], the era reported [16–18] and the landmark used for measuring survival. The extent of improved survival due to individual lines of TKI therapy is unclear. For example, the early imatinib metastatic studies were enriched for patients with bulky disease [4]. Survival in those studies may have been shorter than for metastatic disease patients diagnosed currently with less advanced/bulky disease. Imatinib improves survival when given as adjuvant treatment [19] and the current paradigm of surveillance during and after adjuvant therapy likely leads to the earlier detection of recurrent disease. In addition, the OS reported for the early imatinib studies may not fully capture the additional survival benefits due to use of salvage sunitinib and regorafenib. While sunitinib was initially reported to increase OS compared to placebo [20], the phase 3 study design which allowed crossover from placebo to active drug upon progression, has obscured the actual survival benefit of this agent [20]. The survival benefit of regorafenib is also unclear [21]. Notably, in the phase 3 regorafenib vs. placebo study (GRID), the OS benefit of regorafenib was not identified at the time the results were published in 2012 [21] potentially due to the high rate of cross-over from placebo to regorafenib. Therefore, the impact of 2L sunitinib and 3L regorafenib on the OS of advanced GIST patients remains unclear.

Sunitinib entered clinical testing in 2002 and was FDA approved for 2L treatment of GIST in 2006 [7]. In the phase III trial that resulted in sunitinib regulatory approval, the median time to tumor progression was 6.3 months for sunitinib and 1.5 months for placebo [20]. At the initial publication of trial results, the median OS for those on sunitinib had not been reached but was superior to the placebo group (HR 0.49, 95% CI 0.29–0.83, *p* = 0.007). In a later analysis, the median OS for the sunitinib arm of this trial was 16.7 months [22]. However, the survival difference between the sunitinib arm and the placebo was no longer significant (*p* = 0.306, HR 0.876).

Regorafenib entered phase II testing for GIST in 2010 [23, 24] and phase III studies in 2011 [21], and received FDA approval for IM/SU-resistant GIST in 2013 [8].

Here, we used real-world evidence from patient-reported outcomes to analyze the clinical benefit of 2L sunitinib and 3L regorafenib treatment in advanced GIST OS.

## Methods

### Patient selection

**Fig. 1:**
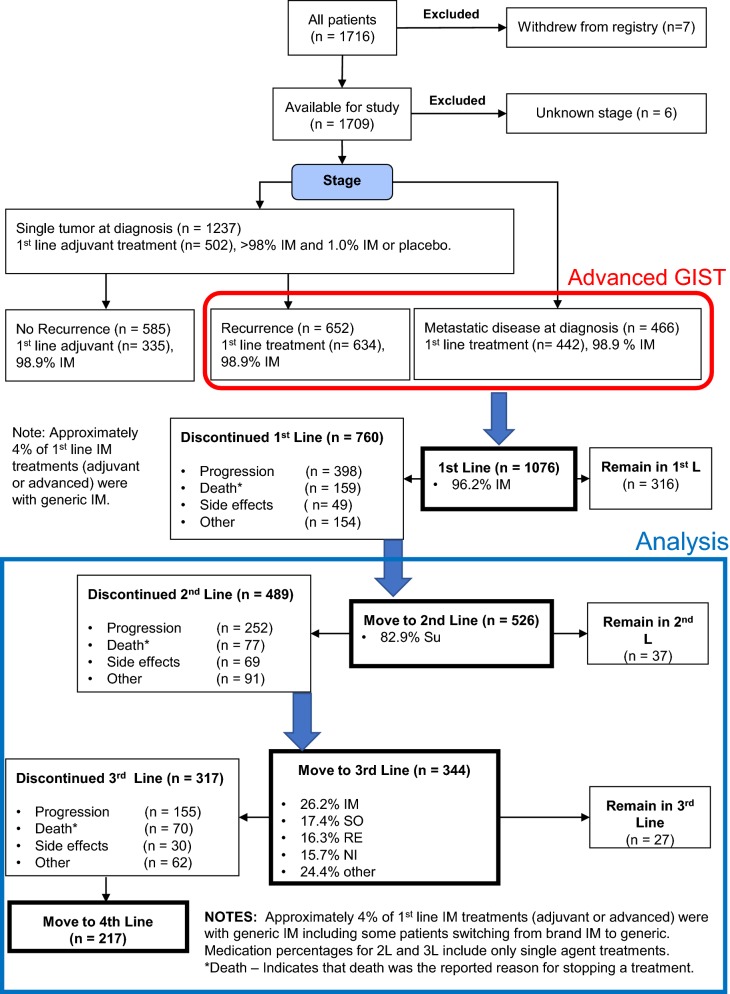
Flowsheet of outcomes as reported by patients

The Life Raft Group Registry started in 2000 and currently includes over 1700 GIST patients (Fig. 1). The Life Raft Group (https://liferaftgroup.org) is an international, internet-based private, non-profit medical research and patient advocacy organization. This is a retrospective analysis of a long-term observational study of those patients.

### Study design and statistical methods

**Fig. 2:**
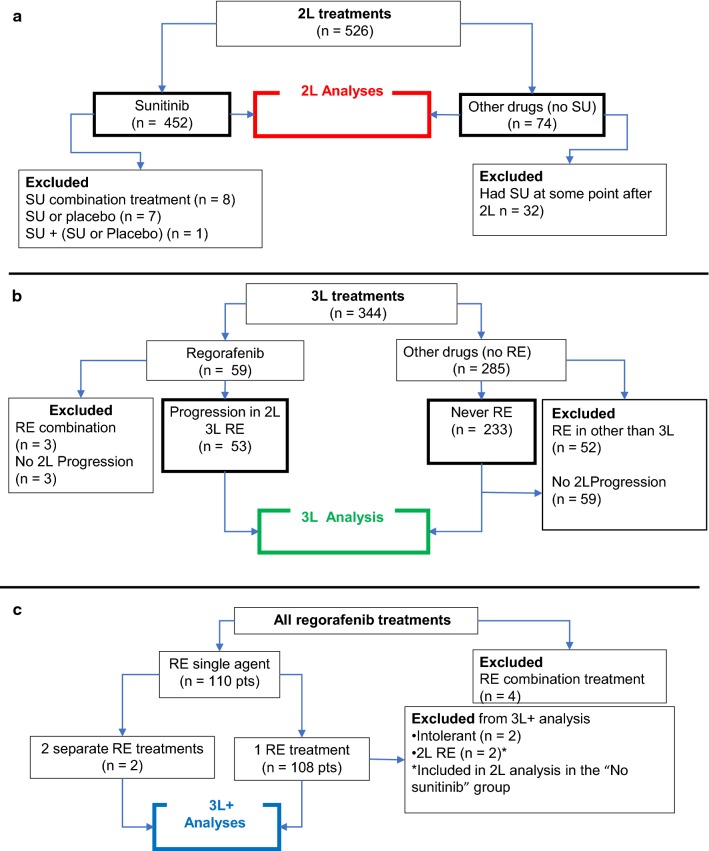
2L, 3L and 3L+ comparison groups

**Fig. 3:**
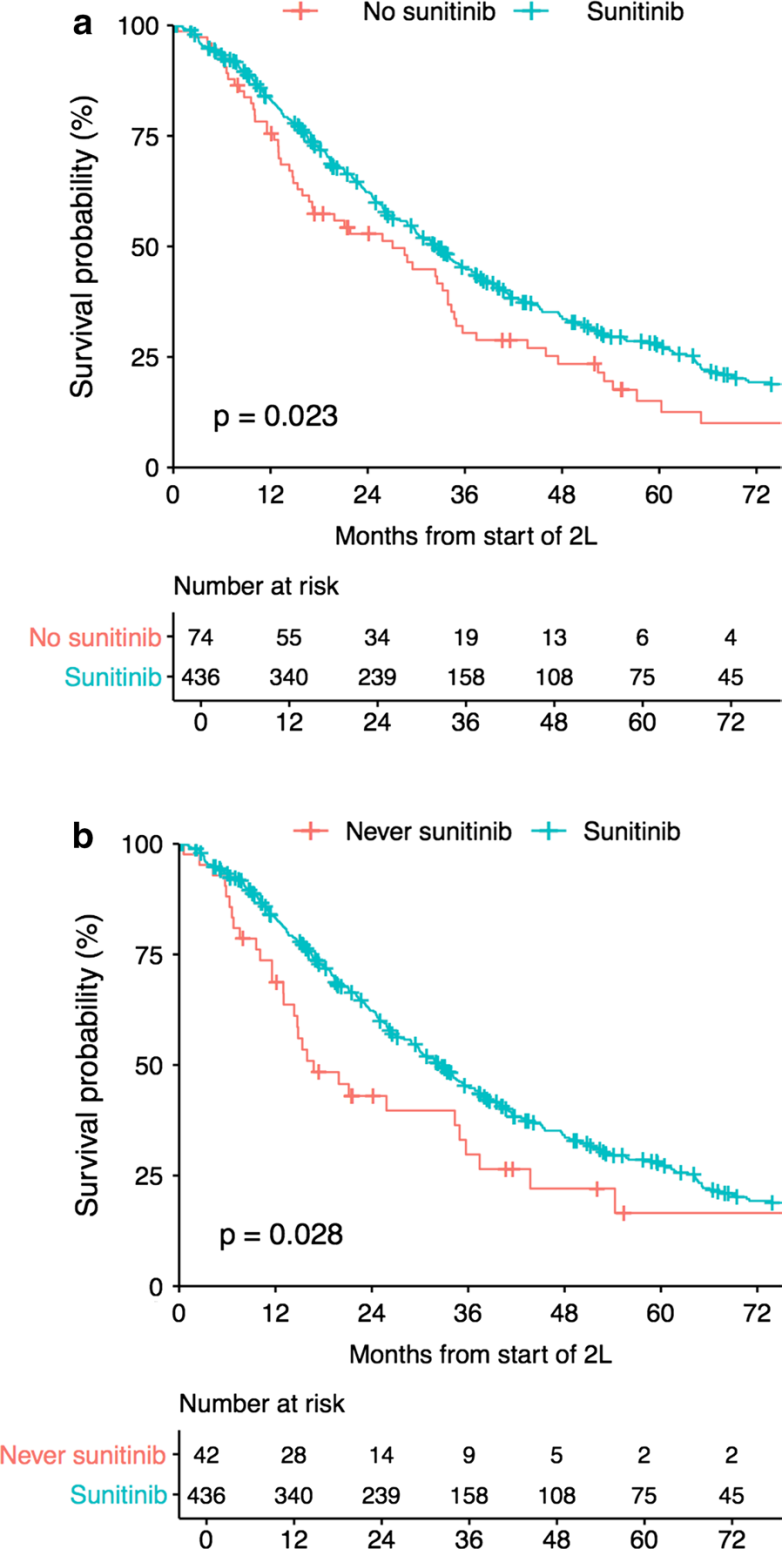
Exploratory analyses of the impact of sunitinib on overall survival. **a** OS 2nd line, all other drugs group *may or may not have had sunitinib later.* **b** OS 2nd line, all other drugs group *excludes patients that had sunitinib at a later time*

**Fig. 4:**
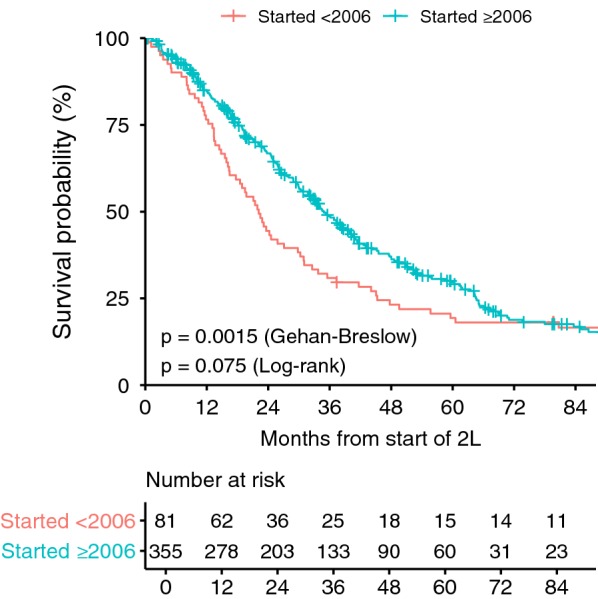
OS 2nd line sunitinib by start year. The Gehan–Breslow–Wilcoxon test gives more weight to early time points and appears to be more appropriate for this analysis because patients in the Start year < 2006 category that live long enough will have increased access to ≥ 3L drugs and thus potential for increased OS

**Fig. 5:**
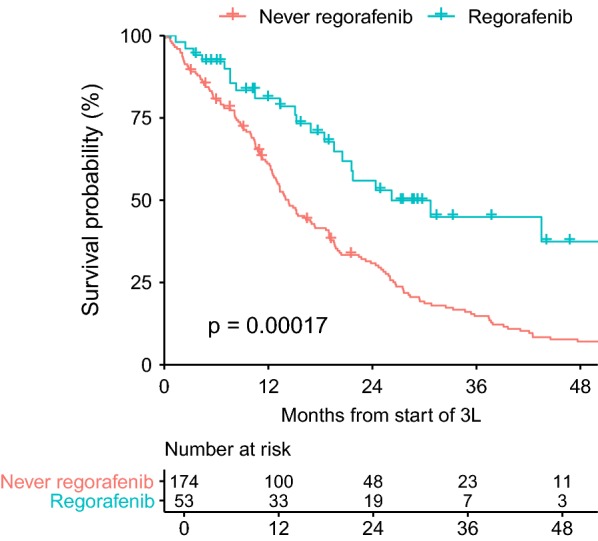
OS 3rd line, all other drugs group *excludes patients that had regorafenib at a later time.* Regorafenib improved overall survival by 11.9 months in 3rd line treatment compared to best supportive care with other TKI’s and excluding no treatment as best supportive care. Patients that had regorafenib in any treatment line were excluded from the all other drugs group. A high percentage of patients in regorafenib group were still alive at last follow-up (censored), suggesting an even greater benefit might be possible. Analysis limited to patients reporting progression in 2nd line

**Fig. 6:**
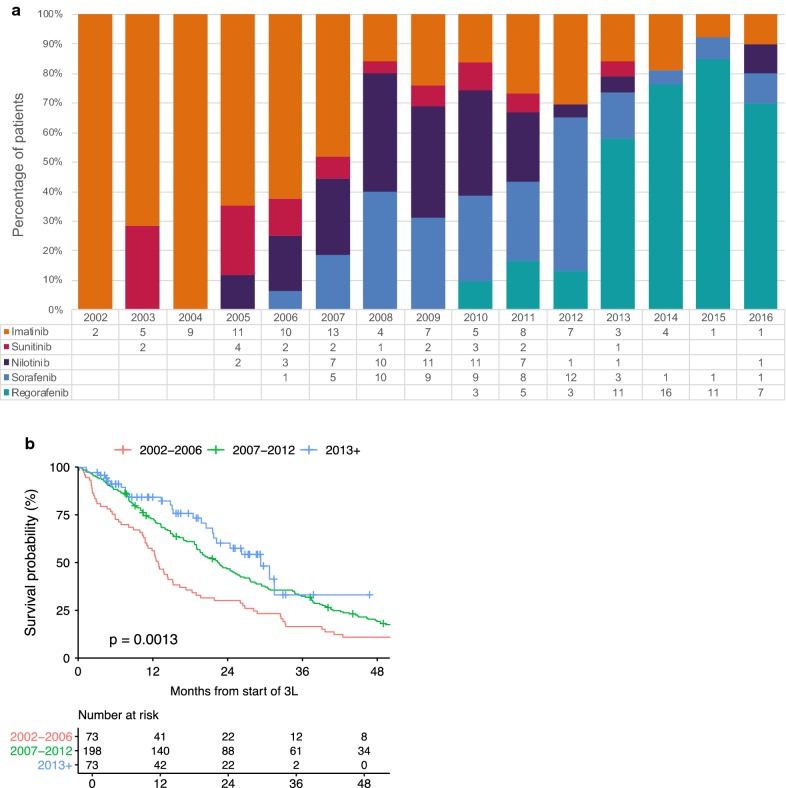
The effect of TKI access over time on overall survival of GIST patients. **a** Most commonly used drugs, 3rd line and beyond by year started 3rd line. **b** OS from start of 3rd line by time period

In this article, we report on OS and self-reported progression-free survival (srPFS) of patients in the registry, with a focus on 2L and 3L treatments (Fig. 2a–c). OS and srPFS estimates were determined using the Kaplan–Meier method and the log-rank test (Figs. 3, 4, 5, 6), or the Gehan–Breslow–Wilcoxon test (Fig. 4). The *p*-values < 0.05 were considered significant. Statistical data analyses were performed using R version 3.5.1 (R Foundation, Vienna, Austria, https://www.R-project.org/). The multi-variable analysis of factors associated with post-2L treatment OS was conducted by Cox regression in IBM SPSS Statistics 25. For the cohort with a variable proportional hazard, we used the Fleming-Harrington weighted log-rank test. The pair–pair variable correlation was analyzed by Spearman correlation coefficiency analysis followed by post hoc Bonferroni correction. For the 3L analysis of the effect of expanded TKI usage over time, patients that reached 3L were divided into three groups based on the year they started 3L treatment (2002–2006, 2007–2012 and 2013+). These categories were selected to coincide with the evolving use of 3L treatments, influenced by approval of new TKIs.

### Treatment lines

Imatinib is the established first line treatment for GIST [9] and was given for > 98% of 1L treatments in the registry. Patient Characteristics are described in Additional file 1: Table S1. The data cutoff date for this study was April 7th, 2017 (Fig. 1). At that time, there were 1716 patients in the registry and 633 were deceased (37%). There are 3249 drug treatments recorded in the registry and 3122 of these were included in our analysis. Excluded treatment records include traditional chemotherapy (often before the TKI era) treatments for a second cancer, known placebo treatments, and instances of intolerance to treatment (defined as discontinuation within 1 month due to side effects). Other than listing the frequency of various first-line treatments, no further analysis of first line treatment is included. Patient characteristics for 3L treatment (which was much more heterogeneous than 2L treatment) is described in Table 1.

**Table 1:** Patient characteristics—3rd line treatment

Year started 3rd line	2002–2006	2007–2012	2013+	Total
Number of patients	73	198	73	344
Alive	3 (4.1%)	27 (13.6%)	48 (65.8%)	78 (22.7%)
Dead	70 (95.9%)	171 (86.4%)	25 (34.2%)	266 (77.3%)
Had regorafenib at any time	2 (2.7%)	48 (24.2%)	61 (83.6%)	111 (32.3%)
Female	31 (42.5%)	77 (38.9%)	39 (53.4%)	147 (42.7%)
Male	42 (57.5%)	121 (61.1%)	34 (46.6%)	197 (57.3%)
Median age/range at start of treatment (3L)	54.0 (18–90)	58.2 (18–88)	60.8 (19–84)	57.8 (18–90)
Age at diagnosis				
< 18	3 (4.1%)	3 (1.5%)	2 (2.7%)	8 (2.3%)
≥ 18–35	9 (12.3%)	21 (10.6%)	6 (8.2%)	36 (10.5%)
≥ 35	60 (82.2%)	174 (87.9%)	65 (89.0%)	299 (86.9%)
Unknown	1 (1.4%)	–	–	
Last reported treatment line				
3L	35 (47.9%)	57 (28.8%)	39 (53.4%)	131 (38.1%)
4L	14 (19.2%)	37 (18.7%)	17 (23.3%)	68 (19.8%)
5L	11 (15.1%)	32 (16.2%)	9 (12.3%)	52 (15.1%)
6L	6 (8.2%)	32 (16.2%)	3 (4.1%)	41 (11.9%)
7L	5 (6.8%)	19 (9.6%)	4 (5.8%)	28 (8.1%)
8L	0	6 (3.0%)	0	6 (11.9%)
9L	2 (2.7%)	8 (4.0%)	1 (1.4%)	11 (3.2%)
10L	0	1 (0.5%)	0	1 (0.3%)
11L	0	3 (1.5%)	0	3 (0.9%)
12L	0	2 (1.0%)	0	2 (0.6%)
13L	0	1 (0.5%)	0	1 (0.3%)
Mutations/% (known mutations)				
KIT	15 (78.9%)	95 (75.4%)	40 (75.5%)	150 (75.8%)
Exon 11	10(52.5%)	64 (50.8%)	31 (58.5%)	105 (53.0%)
Exon 9	5 (26.3%)	25 (19.8%)	7 (13.2%)	37 (18.7%)
Exon 13	–	4 (3.2%)	1 (1.9%)	5 (2.5%)
Exon 17	–	1 (0.8%)	1 (1.9%)	2 (1.0%)
PDGFRA	1 (5.3%)	7 (5.6%	4 (7.5%)	12 (6.1%)
Exon 12		1 (0.8%)	–	1 (0.5%)
Exon 18–D842V	1 (5.3%)	5 (4.0%)	3 (5.6%)	9 (4.5%)
Exon 18 non-D842V		1 (0.8%)	1 (1.9%)	2 (1.0%)
Wildtype for KIT/PDGFRA	3 (15.8%)	22 (17.5%)	7 (13.2%)	32 (16.2%)
SDHA	–		2 (3.8%)	2 (1.0%)
SDHB	–	2 (1.6%)	–	2 (1.0%)
Total/% known mutations	19 (26%)	126 (63.6%)	53 (72.6%)	198 (57.6%)
Unknown mutations	54 (74.0%)	72 (36.4%)	20 (27.4%)	146 (42.4%)
Primary tumor location				
Small intestine	32 (43.8%)	85 (42.9%)	34 (46.6)	151 (43.9%
Stomach	27 (37.0%)	81 (40.9%)	29 (39.7%)	137 (39.8%)
Unknown	7 (9.6%)	10 (5.1%)	2 (2.7%)	19 (5.5%)
Peritoneum	1 (1.4%)	2 (1.0%)	3 (4.1%)	6 (1.7%)
Omentum	1 (1.4%)	3 (1.5%)	1 (1.4%)	5 (1.5%)
Mesentery	1 (1.4%)	4 (2.0%)	–	5 (1.5%)
Rectum/anus	1 (1.4%)	3 (1.5%)	1 (1.4%)	5 (1.5%)
Colon	–	5 (2.5%)	–	5 (1.5%)
Other	3 (4.1%)	5 (2.5%)	3 (4.1%)	11 (3.2%)

The number of patients in each treatment line is described in Additional file 1: Table S2 and single agent regorafenib use in any treatment line is described in Additional file 1: Table S3.

### Patient-reported evaluations and progression

Evaluations including CT scans (84%), MRI’s (6%), PET scans (8%) and ultrasounds (1%) are reported to the registry. Patients have fixed choices to describe the results including (this example is for CT scans), NED, Shrink, Stable, Growth, Mixed and New Tumors. Registrar’s review the results and, in cases of suspected or verified progression, mark the record as progression. In addition, patients can report progression as a reason for changing dose or stopping drug. If progression is recorded in either evaluations or as a reason for stopping/changing drug dose, then the earliest date of progression is recorded and used in srPFS calculations. Radiology reports can be submitted by patients, but most patients do not submit the actual reports. Comments can also be provided by the patients (and are often detailed) in both evaluations and the medication details records.

### Patient-reported surgeries within 2L and 3L treatment periods

Our goal was to evaluate potential benefit of surgery related to 2L and 3L treatment. Since surgery sometimes occurs near a change in treatment, we considered benefit from surgeries occurring just before a treatment change to be most related to the subsequent treatment. As a result, we considered surgery to be within 2L (or 3L respectively) if surgery was done within the period starting 30 days prior to the start of the treatment line and ending 30 prior to the end of the treatment line.

## Results

### Sunitinib as 2L treatment improved patient OS

In the LRG registry, a total of 526 patients received 2L treatment, and 436 patients (82.9%) were treated with single-agent sunitinib (84.6% if sunitinib combination treatments are also counted). For the 436 patients who received single-agent sunitinib as 2L treatment, the median OS from the start of 2L treatment was 32.4 months and the median srPFS was 8.4 months. For the entire group of patients (n = 526) receiving 2L treatments the median OS was 30.3 months.

We performed several exploratory analyses of survival benefit for sunitinib used as 2L treatment. However, because selection bias may play a role in the makeup of the *comparison groups*, the results should be interpreted with caution.

We compared patients that received sunitinib with patients that received another drug as 2L (Fig. 3a). The median OS of the sunitinib group (n = 436) was 32.4 vs. 27.1 months for the alternative 2L treatment group (n = 74, *p* = 0.023, HR 1.377, 95 CI 1.044–1.816). We also compared patients that received sunitinib versus patients that never received sunitinib at any time (Fig. 3b). The median OS of the sunitinib group (n = 436) was 32.4 months vs. 16.8 months for the never received sunitinib group (n = 42, *p* = 0.028, HR 1.52, 95 CI 1.044 to 2.19). The median OS from the start of 2L sunitinib was noted to increase over time (Fig. 4). Patients that started 2L treatment in the time frame the sunitinib studies were recruiting (< 2006), had a median OS of 22.1 months. For patients starting 2L treatment from 2006 to 2016, the median OS was 34.9 months (*p* = 0.0015).

### Factors affecting 2L OS

Using a Cox regression model, we investigated variables affecting 2L OS. The year 2L treatment was started was significant (*p* = 0.029, HR 0.9607) until the use of regorafenib (all except 2 regorafenib treatments were post 2L) was added to the model causing 2L start year to lose significance (*p* = 0.688). In the second model, variables found to be significant were: patient ever had regorafenib (*p* < 0.00001, HR 0.4754), patient reported progression in 1L (*p* = 0.0008, HR 1.7121, favoring those not reporting progression in 1L), patient ever had sunitinib (*p* = 0.022, HR 0.6477), patient reported knowing their mutation (*p* = 0.0001, HR 0.6522, favoring the patients that knew their mutation), gender (*p* = 0.0004, HR 1.4658, favoring females) and age at start of treatment (p < 0.00001, HR 1.0203 per year).

In the third and final model (Additional file 1: Table S4), when patients knowing or not knowing their mutation was replaced with the actual mutation (but still including unknown mutation as a group) only two groups were significantly different than patients with KIT 11 mutations (the reference mutation). Patients with no mutation in KIT or PDGFRA (KIT/PDGFRA WT) had longer 2L OS (p = 0.0048 HR 0.4830), and patients reporting as unknown mutation had shorter 2L OS (p = 0.037 HR 1.3194). Of the 12 D842V 2L patients, 9 had treatment at some time (not necessarily in 2L) with one of four agents with suspected activity against D842V mutations, dasatinib [25] (n = 5 pts), crenolanib [26] (n = 5 pts), avapritinib [27] (BLU-285, n = 3) or olaratumab [28] (n = 2). Although the hazard ratio of 0.5799 for D842V patients compared to KIT exon 11 patients was relatively low, the difference in 2L OS was not significant, *p* = 0.1691, however in multi-variable analysis it was significant in 3L, provided start year was used as a variable instead of start year category (p = 0.024 HR 0.3484). The small number of D842V-directed treatments spread across different treatment lines limits the efficacy analysis of any specific treatment for patients with the D842V mutation. In addition, the trajectory of the disease seems to have more interpatient variability in the KIT/PDGFRA WT and D842V groups, with some of these patients having a more indolent course. In the KIT/PDGFRA WT group this is likely due to a high percentage of undiagnosed SDH-deficient patients within the group and their known potential for an often indolent nature [29]. This interpatient variability combined with small numbers of patients and the limitations of the median as a sole outcome measure (especially with small numbers) can sometimes lead to counterinitiative results. For example, in this series with 3 patients failing to transition from 2L to 3L, the 3L D842V patients (n = 9, median OS 61.7 months), had longer OS than 2L D842V patients (n = 12, median OS 34.4 months).

### Regorafenib treatment improves OS

In the LRG registry, 114 patients received regorafenib in 117 separate treatments (3 pts received two separate treatments each with regorafenib and one of these 6 treatments was a combination treatment). One hundred and seven patients receiving 109 separate treatments are included in the 3L+ analyses. See Additional file 1: Table S3 for regorafenib usage by treatment line.

Fifty-six of these patients received single-agent treatment in the 3L, matching the current approved indication; three additional patients received regorafenib in combination with embolization in 3L for a total of 59 3L treatments. The three regorafenib combination treatments were excluded from analysis.

In the 3L, 53/56 (94.7%) patients on regorafenib reported progression in 2L. In addition, 285 patients received a drug other than regorafenib for 3L treatment and 233 of these patients never received regorafenib at any time. Further analysis was restricted to those that never received regorafenib in any line of therapy. Comparing these two groups, the median OS was 26.2 months for the regorafenib group and 15.2 months for the never received regorafenib group (*p* = 0.0027). In the 233 patients treated with a drug other than regorafenib who never received regorafenib in any line of treatment, 174 (74.7%) of 233 reported progression in 2L. Due to this imbalance compared to those that received regorafenib 3L, we restricted further analyses to patients that reported progression in 2L.

For 3L treatment of patients *reporting progression in 2L*, 53 received regorafenib and 174 never received regorafenib. Between these groups, regorafenib treatment compared with never receiving regorafenib was associated with improvement in OS by 11.9 months (26.2 months vs. 14.3 months, respectively, *p* = 0.0002, HR 2.231, CI 1.45–3.43) (Fig. 5). The most common drugs used in 3L in the no regorafenib/progressed in 2L group were imatinib (n = 50, 28.9%), nilotinib (n = 37, 21.4%), sorafenib (n = 36, 20.8%) and sunitinib (n = 10, 5.8%). The use of sorafenib and nilotinib was likely influenced by ongoing phase 2–3 studies of these agents prior to the approval of regorafenib [30–33].

One hundred and seven LRG registry patients received 109 treatments with regorafenib monotherapy beyond 2L (two patients received regorafenib in two separate treatment lines). The median OS of these patients was 22.5 months and the median srPFS was 7.2 months (Table 2). Fifty-six (51.4%) of these patients received regorafenib 3L and 53 (48.6%) received regorafenib after 3L. These numbers are similar to the GRID study (Table 2), where 44% received regorafenib after 3L [21]. In that study, 133 patients in the regorafenib arm had an OS of 17.4 months [22] and PFS of 4.8 months [21], however, investigator-assessed median PFS was 7.4 months.

**Table 2:** LRG registry 3L+ regorafenib use compared to GRID trial

	No. of Pts/treatments	> 3rd line %	Median OS (months)	Median PFS/srPFS (months)
Phase III GRID Trial (regorafenib arm only)	133	44	17.4^32^	4.8^a^
LRG Registry 3L+	107/109^b^	48.6	22.5	7.2

^a^7.4 investigator assessment

^b^Two patients received two separate treatments of regorafenib beyond 2L for a total of 109 separate treatments beyond 2L. Four other patients received regorafenib 2L for a total of 113 treatments with single-agent regorafenib in the registry. Two of the 2L patients were intolerant to regorafenib. Four other patients received regorafenib in combination with another treatment. A total of 114 patients received 117 treatments with regorafenib when all treatment lines, single agent and combinations were included

### The effect of continued TKI access in patients in the pre-regorafenib era and into the regorafenib era

Third line treatment in the LRG registry was much more varied than 1L or 2L and was highly correlated with the time period at the start of 3L therapy (Fig. 6a and Additional file 1: Table S5). The participation in clinical studies of sunitinib and regorafenib, the approval of sunitinib for 2L in 2006, regorafenib for 3L in 2013 and the availability of nilotinib and sorafenib starting in ~ 2007 (either off-label or in phase 2–3 studies) influenced treatment patterns for advanced GIST (Fig. 6a). From 2002 through 2006, treatment with imatinib dominated 3L and 3L+ treatments. 2007 was a transition year between imatinib use and subsequent higher rates of nilotinib or sorafenib usage. This trend continued in 2008 with high usage of either nilotinib or sorafenib, and some continued use of imatinib or sunitinib in later treatment lines. In 2012, use of nilotinib dropped while sorafenib use remained common through 2012, dropping with the approval of regorafenib in 2013. From 2013 through 2016, regorafenib was the dominant 3L treatment.

Measuring the impact of survival due to later treatment lines, can be confounded by the small numbers of patients for any given treatment line, the number of different drugs used for these treatments and the impact of additional later treatments. To correct for this, we investigated the *cumulative* impact of drugs by looking at the *time period when they were used* and also measuring OS from a common time point: the start of 3L treatment.

We defined 3 patient cohorts, grouped by the year in which 3L treatment began to match the major shifts in treatment patterns over time (Fig. 6a and Additional file 1: Table S5). The first group started 3L treatment between 2002 and 2006 (73 patients). The second group started 3L between 2007 and 2012 (198 patients) and the third group started 3L in 2013 or later (73 patients).

As shown in Fig. 6b in the 2002–2006 group, characterized by the dominant use of imatinib in 3L or later, the median OS from the start of 3L therapy was 12.8 months. Although imatinib was the dominant 3L drug in this period, 52% of patients from this period received additional treatments beyond 3L (Table 1). In the 2007–2012 group characterized by the frequent use of nilotinib or sorafenib, the median OS was 22.4 months. In the 2013+ group, characterized by the dominant use of 3L+ regorafenib, the median OS was 29.3 months.

### Factors affecting 3L OS

Using a Cox regression model, we investigated variables affecting OS as measured from the start of 3L treatment. Of the variables found to be significant in univariable analysis (Additional file 1: Table S6), only the time period 3L treatment began lost significance in the initial multivariable analysis. Variables maintaining significance in the initial multi-variable analysis were: patient-reported progression in 2L (*p* = 0.001, HR 1.698), patient treated with regorafenib at any time (*p* < 0.0001, HR 0.441), age at start of treatment (*p* < 0.0001, HR 1.020), gender (*p* = 0.041, HR 0.769, favoring OS in females) and whether or not the patient had a known mutation type (*p* = 0.011, HR 0.715, favoring known mutation). When we replaced the known/unknown variable with the actual mutation the results were similar (Additional file 1: Table S7) with the exception of gender and unknown mutation (as a category in the mutation variable) which both lost significance (*p* = 0.14 and *p *= 0.192).

These results suggest that the use of nilotinib and/or sorafenib in 3L or 3L+ treatment lines improved survival over imatinib, and that the use of 3L regorafenib was associated with improved OS compared with use of nilotinib and/or sorafenib.

### Exploratory analysis of treatment choice and performance status

In some cases, poor performance status may affect treatment choice. For example, patients with poor performance status might choice imatinib therapy instead of sunitinib or regorafenib if they believed imatinib was more tolerable. If this was common, it might reduce both PFS (or srPFS in the case of the LRG registry) and OS of groups with more imatinib patients. The LRG registry does not track performance status or tumor burden.

To explore the relationship of treatment choice, performance status and OS we investigated imatinib use in three areas; the reason that patients stopped treatment in the previous treatment line, the percentage of patients starting 3L with imatinib compared to nilotinib or sorafenib and the survival of 3L patients starting imatinib versus those starting 3L nilotinib or sorafenib.

As our main comparison group, we used 3L treatments where patients started treatment between 2007 and 2012. We chose this group because it had the greatest heterogeneity in terms of both drug use and choice of treatment options available.

In the patients starting 3L treatment between 2007 and 2012, patients without progression in 2L treatment (no report of progression from an evaluation or as reason for stopping treatment) choose 3L imatinib (compared to nilotinib and sorafenib) more often than patients reporting 2L progression (51% versus 24%). In the 2007 and 2012 group, patients listing side effects as reason for stopping 2L treatment choose 3L imatinib (30% of 3L imatinib patients) more often than other drugs for 3L treatment (13% of nilotinib patients and 11% of sorafenib patients) and patients choosing 3L imatinib within the time frame of maximum choice (2007–2012) had equal survival to those choosing 3L nilotinib/sorafenib (*p* = 0.527).

### Exploratory analysis of surgery

Since it has been suggested that surgery for patients with metastatic GIST may be beneficial [34–36], we investigated the impact of surgery within 2L and 3L treatments. Given the limitations of retrospective data in evaluating surgical benefit (see Surgery and Surgery Limitations in the “Discussion” section), the first question that we were interested in was whether surgery was equally balanced in the different groups within our study. In 2L treatment, surgery was well-balanced with 14.5% of sunitinib patients having surgery within 2L and 9.5% of patients never receiving sunitinib having surgery. The percentage of patients have surgery in third line treatment was also well-balanced with respect to 3L treatment start date categories (2002–2006 12.3%, 2007–2013 6.6% and 2013+ 9.6%) and patients having 3L regorafenib (9.9% had surgery) or never having regorafenib (7.7% had surgery).

Patients having surgery within both 2L and 3L had significantly better 2L or 3L OS than patients not having surgery within those treatment lines. The median OS in 2L was 66.1 months for patients having 2L surgery (n = 81) versus 32.4 months for patients not having 2L surgery (n = 445); *p* = 0.0008, HR 0.56 CI 0.42–0.64). The median OS in 3L was 37.4 months for patients having 2L surgery (n = 36) versus 19.7 months for patients not having 2L surgery (n = 308) [*p* = 0.0008, HR 0.40, 95 CI 0.25–0.75].

Patient-reported options for reporting the reason for surgery were: metastasis, primary tumor, primary tumor and metastasis, local recurrence and other. In patients reporting 2L or 3L surgery, only surgeries that involved a primary tumor (primary tumor or primary tumor with metastases) separated significantly from other reasons, however this type surgery was rare in 2L (n = 9) and involved only 2 cases in 3L.

Interestingly, given the option to choose Clear Margins, No Clear Margins or Not Known, the No Clear Margins group had worse OS in both 2L and 3L. For 2L patients, the median OS was, Clear Margins (n = 23) 61.3 months, No Clear Margins (n = 18) 21.4 months and Not Known (n = 40) 63.4 months. Compared to Clear Margins, the No Clear Margins patients had increased risk of death, HR 3.87 (CI 1.79–8.34), *p *= 0.0056).

## Discussion

Imatinib-resistant GIST remains challenging to treat. However, OS has improved significantly over time. This appears to be primarily due to increased access to new treatments; especially sunitinib and regorafenib, but also the frequent use of nilotinib and sorafenib in this patient population prior to regorafenib availability.

The very short PFS in patients receiving placebo in the randomized sunitinib [20] or regorafenib clinical studies [21] validates the long-standing hypothesis that stopping TKI treatment in the setting of TKI-resistant GIST results in rapid disease progression. In fact, a randomized study demonstrated that imatinib re-challenge of patients with imatinib- and sunitinib-resistant GIST improved PFS compared with placebo [37]. Therefore, use of TKIs such as nilotinib and sorafenib might act similarly; controlling imatinib-sensitive and some, but not necessarily all, imatinib and/or sunitinib resistant clones with secondary mutations, thus providing a benefit after imatinib/sunitinib failure.

The initial report [20] showed an OS benefit for sunitinib compared to placebo in a phase III clinical trial; however, in the final analysis in 2012 [38] there was no longer an OS benefit (*p* = 0.306). The high rate of cross-over from placebo to sunitinib obscures the survival benefit. It is not possible to determine the actual survival benefit with this trial design which is also the same design used in the GRID trial for regorafenib. The short time off of medication combined with the ability to access additional treatments obscures the actual benefit. Interestingly, patients in the LRG registry showed a similar pattern with respect to time on 2L treatment. Patients starting 2L prior to 2006 initially had poorer survival than patients that started 2L ≥ 2006 (Fig. 4), however the survival curves eventually cross after about 6 years. It’s tempting to speculate that the long-term survivors might have similarities that allow patients in both groups to survive long-term in spite of different initial treatments. An example would be SDH-deficient patients which, in general, are known to have a more indolent course of disease [29].

Herein, we report a real-world estimate of median OS for 2L sunitinib from the start of treatment of 32.3 months and a median srPFS of 8.4 months. Both of these survival estimates, especially OS, are longer than previous reports. PFS or Time to Progression (TTP) times reported by investigator assessment in clinical studies are often longer than those reported using blinded central radiology review (e.g. GRID [21]). Notably, our registry srPFS time reported here (8.4 months) is almost identical to the investigator reported TTP (8.3 months) in a very large (1124 patients) treatment use trial reported by Reichardt et al. [39].

We investigated the reasons for longer OS of the LRG registry patients receiving 2L sunitinib and 3L regorafenib compared to data from clinical studies and other reports. One significant finding is that OS has improved over time, so our registry population benefits from including patients from later time periods, especially compared to the time period when the phase III sunitinib trial was conducted. For example, if we compare the median OS for LRG patients starting 2L sunitinib prior to 2006 (Fig. 4) to the phase III clinical study which enrolled patients from December, 2003 until January, 2005, the median OS is much closer, 22.1 (LRG) vs 16.8 months (phase III study). Another factor is that a higher percentage of LRG patients starting 2L did not have documented progression in 1L compared to patients in the clinical studies. In our series, 374 of the 436 (86%) of 2L sunitinib-only patients reported progression with 1L treatment. The OS from the start of 2L between patients reporting progression in 1L and patients not reporting progression in 1L differed substantially, 30.8 months vs. 45.5 months respectively (p = 0.0055, HR 1.597). In our regorafenib analyses, we focused on 3L treatment; in the case of the GRID study, patients were not limited to 3L and 44% of the regorafenib group (59 of 133) had > two lines of previous therapy. As expected, median OS decreases with each line of therapy.

An area of active investigation by our group is whether proactive, educated, engaged patients, such as those who are represented in the LRG registry, have better outcomes than less engaged patients. For example, registry patients may more frequently seek out additional treatments after failure of standard treatments, including clinical studies and off-label treatments. This hypothesis is supported by the high percentage of patients undergoing treatment beyond 3L. Patients in the LRG registry may more frequently consult with GIST experts; possibly resulting in greater access to additional therapies, including investigational agents.

In regard to the hypothesis that patients with poor performance status might choose imatinib in later treatment lines. We cannot exclude that this is the primary reason that *some* patients choose imatinib; however, our data suggests that the number choosing imatinib for this reason may be fairly small and may not significantly impact OS. In fact, using 3L as an example, we found the opposite case was more likely with a higher percentage of 3L patients not reporting progression in 2L choosing imatinib (51% of imatinib patients starting 3L between 2007 and 2012) compared to patients reporting progression in 2L (24% of 3L patients staring between 2007 and 2012). It is likely that a factor other than drug choice is more indicative of patients with very poor performance status. This factor is how many patients successfully transition to the next treatment line. For patients in the LRG registry, about 30% (range 27.3–30.7%) of patients fail to move to the next treatment line at each stage from the 2L to 3L transition until the 5L to 6L transition rising to about 50% at the 6L to 7L transition. The percentage of patients able to successfully transition to the next treatment line is related to both performance status (with the sickest patients unable to transition to the next treatment line) and availability/access to additional treatment options, which varied over time.

## Limitations

Life Raft Group registry members are self-referred. Low-risk patients are less likely to participate, and the LRG registry has a higher percentage of high-risk patients and patients with metastatic disease at diagnosis compared to population-based studies [1, 40]. Younger patients are more likely to be internet/technology savvy than older patients and thus more likely to participate in the registry. Proactive patients may also be more likely to participate in the registry as well as more likely to seek treatment from GIST expert centers and to participate in clinical trials. Lack of internet access, language barriers and social/economic status are also likely barriers to participation. In the vast majority of cases, patients reported as not knowing their mutation, did not have a mutational test performed. However, in some cases, it’s possible that a mutational test was performed without the patient’s knowledge or understanding of the test and it’s also possible that some patients failed to report the test to the registry.

In our opinion, the patients in the second line *comparison arms* were more likely to be subject to selection bias compared to the third line patients. This is because sunitinib was generally available for almost all of the 2L patients, but for 3L patients, the treatments were largely selected by which treatment patients were able to access in different time periods. In some cases, there may have been reasons that a patient selected a drug other than sunitinib for 2L, for example, two patients with mutations known to be sunitinib-insensitive (D842V mutations) selecting crenolanib (a PDGFRA inhibitor with activity against the D842V mutation) instead of sunitinib for 2L. For this reason, the comparisons of other drugs compared to sunitinib in 2L should be interpreted with caution. This selection bias is more likely to affect the comparison groups than the sunitinib groups.

Five and a half percent of registry patients had a combination treatment. Combination treatments were more common after second line, with 11.4% of patients receiving a combination treatment when treatment lines beyond 2L were combined. In both 2L and 3L, patients receiving a combination treatment did somewhat better than patients not receiving a combination, however the difference was not significant. To generate the most conservative estimate of benefit when comparing treatments, combination treatments were excluded from the sunitinib (2L) and regorafenib (3L) groups but included in their respective comparison groups. These comparison groups are essentially best alternative treatments (physician’s choice) including combinations.

### Surgery and surgery limitations

The limitations of retrospective data to evaluation surgical benefit for metastatic GIST have been well-described in the literature [34–36]. A primary concern is that healthier patients are often better surgical candidates. In addition to these well-described limitations, the data structure in the LRG registry was not designed to identify generalized progression versus local progression or stable metastatic disease at the time of surgery. Given these limitations, we can only state that patients that had surgery in 2L or 3L had longer OS than those that did not, but we cannot conclude whether this was due to surgical benefit or because they were healthier patients. Failure to obtain clear margins in 2L or 3L surgery was also associated with significantly worse OS. Since the term “clear margins” is more appropriately used to describe surgical margins after primary tumor therapy, it is necessary to consider how patients might interpret it in the context of 2L or 3L surgeries. In this context, it is more likely to be interpreted by patients and registrars as no evidence of disease after surgery. Interestingly, when given the choice of Clear Margins, Not Known or No Clear Margins, Clear Margins and Not Known grouped closer together (especially in 2L) and the No Clear Margins patients had clearly worse OS.

Given the limitations of retrospective surgery data, our primary concern was whether surgery might influence our primary medical treatment analysis if surgery was unbalanced across treatment groups. This did not appear to be the case. Also, given the limitations, we did not include surgery in the multi-variable analysis.

## Conclusions

The real-world data reported here strongly support the hypothesis that sunitinib improves OS in advanced GIST patients. It also suggests that use of TKI’s in the 3L, especially nilotinib and sorafenib improved OS in the period prior to regorafenib approval. Regorafenib use was common in the 2013+ period and was associated with significantly improved survival of patients compared to the time periods characterized by dominant 3L imatinib use (2002–2006) or 3L+ use of nilotinib and/or sorafenib (2007–2012). In a multi-variable analysis of OS from start of 3L, patients that received regorafenib at any time had a 61% reduction in the risk of death compared to those that never had regorafenib at any time (Additional file 1: Table S7). Interestingly, using Cox regression analysis, we also show a positive impact on 2L OS of a drug taken after 2L (regorafenib), something that is not possible with traditional clinic trial designs. When citing or referring survival times, it’s important to understand that survival may have changed over time and referencing older data may be misleading. This is especially important to keep in mind when talking to patients.

These data support current clinical guidelines for treatment of advanced disease. Our data also suggest that continued development of effective TKIs may further improve the treatment of advanced GIST. In addition, our data support the use of patient reported data in addressing outcome questions that cannot be addressed in single arm interventional studies or by randomized studies of investigational agents vs. placebo that incorporate a cross-over design.

## Additional file

**Additional file 1.** Additional tables.

## Abbreviations

2L: 2nd line
3L: 3rd line
FDA: Federal Drug Administration
GIST: gastrointestinal stromal tumors
HR: hazard ratio
ICC: interstitial cells of Cajal
IM: imatinib
IRB: Institutional Review Board
KIT/PDGFRA WT: wildtype for both *KIT* and *PDGFRA* genes
LRG: Life Raft Group
NI: nilotinib
OS: overall survival
Pt: patient
RE: regorafenib
SO: sorafenib
srPFS: self-reported progression-free survival
SU: sunitinib
TKI’s: tyrosine kinase inhibitors
TL: treatment line
WT: wildtype
