# Correction to: Survival in advanced GIST has improved over time and correlates with increased access to post-imatinib tyrosine kinase inhibitors: results from Life Raft Group Registry

**DOI:** 10.1186/s13569-019-0117-2

**Published:** 2019-05-07

**Authors:** Jerry W. Call, Yu Wang, Denisse Montoya, Norman J. Scherzer, Michael C. Heinrich

**Affiliations:** 1 Life Raft Group, 155 Route 46 West, Suite 202, Wayne, NJ 07470 USA; 2 Portland VA Health Care System and Knight Cancer Institute, Oregon Health & Science University, Portland, OR USA

## Correction to: Clin Sarcoma Res (2019) 9:4 10.1186/s13569-019-0114-5

The legends for Figs. 3 and 5 of the article [1] incorrectly refer to an “all others group”. The corrected legends should read:

**Fig.** **3** Exploratory analyses of the impact of sunitinib on overall survival. **a** OS 2nd line, the “No sunitinib” group *may or may not have had sunitinib later.* **b** OS 2nd line, the “Never sunitinib” group *excludes patients that had sunitinib at a later time.*

**Fig.** **5** OS 3rd line, the “Never regorafenib” group excludes patients that had regorafenib at a later time. Regorafenib improved overall survival by 11.9 months in 3rd line treatment compared to best supportive care with other TKI’s and excluding no treatment as best supportive care. Patients that had regorafenib in any treatment line were excluded from the “Never regorafenib” group. A high percentage of patients in “Regorafenib” group were still alive at last follow-up (censored), suggesting an even greater benefit might be possible. Analysis limited to patients reporting progression in 2nd line.
