# Supplementary material for: Survival in advanced GIST has improved over time and correlates with increased access to post-imatinib tyrosine kinase inhibitors: results from Life Raft Group Registry
Source: Clin Sarcoma Res. 2019 Apr 2;9:4. doi: 10.1186/s13569-019-0114-5 (PMC6446260; doi:10.1186/s13569-019-0114-5)
Supplement: Supplementary file 1 — Additional file 1. Additional tables. [file 13569_2019_114_MOESM1_ESM.docx]

**Additional Tables**

| Table S1 – Mutational Testing and Patient Characteristics of Registry | | | |
| --- | --- | --- | --- |
|  | Known Mutation | Unknown Mutation | Total  (% of total) |
| Patients (all) | 739 (43.2%) | 970 (56.8%) | 1709 |
| Female | 392 (53.0%) | 465 (47.9%) | 857 (50.1%) |
| Male | 347 (47.0%) | 505 (52.1%) | 852 (49.9%) |
| Alive | 544 (50.6%) | 532 (49.4%) | 1076 (63.0%) |
| Deceased | 195 (30.8%) | 438 (69.2%) | 633 (37.0%) |
| Age at diagnosis category |  |  |  |
| <18 | 23 (56.1%) | 18 (43.9%) | 41 (2.4%) |
| 18-35 | 81 (49.1%) | 84 (50.9%) | 165 (9.7%) |
| >35 | 634 (42.4%) | 863 (57.6%) | 1497 (87.6%) |
| Unknown | 1 (16.7%) | 5 (83.3%) | 6 (0.4%) |
| Median | 50.7 yrs | 51.4 yrs | 51.1 yrs |
| Diagnosis year |  |  |  |
| <2000 | 58 (31.2%) | 128 (68.8%) | 186 (10.9%) |
| 2000-2006 | 266 (35%) | 495 (65%) | 761 (44.5%) |
| 2007-2012 | 335 (52.2%) | 307 (47.8%) | 642 (37.6%) |
| 2013-2017 | 80 (67.2%) | 39 (22.8%) | 119 (7.0%) |
| Stage at Diagnosis |  |  |  |
| Single Tumor at Diagnosis | 511 (42.7%) | 684 (57.2%) | 1195 (70.0%) |
| Mets at Diagnosis | 204 (43.8%) | 262 (56.2%) | 466 (27.3%) |
| Multi-focal at diagnosis | 22 (55%) | 20 (45%) | 42 (2.5%) |
| Unknown | 2 (33.3%) | 4 (66.7%) | 6 (0.4%) |
|  |  |  |  |
| Had Adjuvant | 278 (54.9%) | 228 (45.1%) | 506 (29.6%) |
| 1M treatment | 457 (42.5%) | 619 (57.5%) | 1076 (63%) |
| 2000-2005 | 160(28.9%) | 393 (71.1%) | 553 (51.4%) |
| 2006-2012 | 244 (54.6%) | 203 (45.4%) | 447 (41.5%) |
| 2013-2017 | 53 (69.7%) | 23 (30.3%) | 76 (7.1%) |
| 2L treatment |  |  |  |
| 2002-2006 | 52 (27.7%) | 136 (72.3%) | 188 (35.7%) |
| 2007-2012 | 154 (61.1%) | 98 (38.9%) | 252 (47.9%) |
| 2013-2017 | 61 (70.9%) | 25 (29.1%) | 86 (16.3%) |

| Table S2 - Treatments by Treatment Line | |
| --- | --- |
| Treatment Line | No. of Treatments |
| 1M* | 1076 |
| 1A † | 506 |
| 1N‡ | 102 |
| 2 | 526 |
| 3 | 344 |
| 4 | 213 |
| 5 | 144 |
| 6 | 92 |
| 7 | 52 |
| 8 | 24 |
| 9 | 18 |
| 10 | 7 |
| 11 | 6 |
| 12 | 3 |
| 13 | 1 |
| Other | 135 |
| *1M = 1L treatment for advanced/metastatic GIST  †1A = 1L adjuvant treatment  ‡1N = 1L neoadjuvant treatment | |

| Table S3 – Single agent regorafenib use in LRG registry | | | |
| --- | --- | --- | --- |
| Treatment Line | No. of patients | Median OS (mo.) | Median srPFS |
| 2 | 2 | N/R | N/R |
| 3 | 56 | 26.2 | 9.0 |
| 4 | 24 | 25.4 | 7.3 |
| 5 | 11 | 13.3 | 7.0 |
| 6 | 5 | 14.4 | Undefined |
| 7 | 5 | 19.8 | 6.1 |
| 8 | 1 | N/R | N/R |
| 9 | 6 | 13.0 | 14.5 |
| 13 | 1 | N/R | N/R |
| Total (combined) | 111 | 22.5 | 7.2 |
| 2 INT* | 2 | N/R | N/R |
| *intolerant, discontinued within 1 month  N/R = Not Reported (due to small numbers)  Note: Four patients receiving regorafenib in combination with another treatment were excluded from this table (three embolizations and one combination of sunitinib and regorafenib). | | | |

| Table S4– Multi-variable analysis – OS from start of 2L, Cox regression | | | | |
| --- | --- | --- | --- | --- |
|  | p value | Hazard Ratio | 95% CI for HR | |
|  |  |  | Lower | Upper |
| **Start Year** | 0.514 | 0.9874 | 0.9503 | 1.0258 |
| **Patient Reported Progression in 1^st^ Line** |  |  |  |  |
| Yes | - | 1 | - | - |
| No | 0.001 | 0.5948 | 0.4337 | 0.8157 |
| **Ever Had Sunitinib** |  |  |  |  |
| No | - | 1 | - | - |
| Yes | 0.0257 | 0.6531 | 0.4491 | 0.9497 |
| **Ever Had Regorafenib** |  |  |  |  |
| No |  | 1 |  |  |
| Yes | <0.001 | 0.4668 | 0.314 | 0.620 |
| **Age at Start of Treatment (per year)** | <0.001 | 1.0159 | 1.074 | 1.0245 |
| **Gender** |  |  |  |  |
| Male | 0.043 | 1.3691 | 1.1031 | 1.6992 |
| Female | - | 1 | - | - |
| **Mutation** |  |  |  |  |
| KIT exon 11 | - | 1 | - | - |
| KIT exon 9 | 0.880 | 0.9708 | 1.0301 | 1.4245 |
| D842V (PDGFRA, exon 18) | 0.169 | 0.5799 | 0.2667 | 1.2608 |
| KIT/PDGFRA WT | 0.005 | 0.4830 | 0.2911 | 0.8013 |
| Rare KIT/PDGFRA mutations combined, (KIT exons 13,17, PDGFRA exons 12, 14) | 0.397 | 0.7150 | 0.6431 | 3.0413 |
| Unknown mutation | 0.0368 | 1.3194 | 1.0171 | 1.7115 |
|  |  |  |  |  |

| Table S5 **–** Major Drugs Used ≥ 3L by Year Patient Started 3^rd^ Line | | | | |
| --- | --- | --- | --- | --- |
|  | **2002-2006** | **2007-2012** | **2013+** | **Total** |
| **Number of patients – start of 3L** | 73 | 198 | 73 | 344 |
| ***Regorafenib** | 2 | 48 | 61 | 111 |
| ***Sorafenib** | 10 | 115 | 9 | 134 |
| ***Nilotinib** | 21 | 94 | 79 | 122 |
| ***Imatinib** | 56 | 93 | 23 | 172 |
| Patient had at least 2 of the 4 drugs given in different treatment lines ≥ 3^rd^ line | 16 | 84 | 13 | 113 |
| None of the 4 drugs above | 10 | 11 | 3 | 24 |
| *Had drug as single agent or a combination (e.g., imatinib + embolization), at any time ≥3L. | | | | |

| Table S6- Univariate Analysis – OS from start of 3L, Cox Regression | | | | |
| --- | --- | --- | --- | --- |
| Variable | *p* value | Hazard Ratio | 95% CI of HR | |
|  |  |  | Lower | Upper |
| **Start Category** |  |  |  |  |
| 2002-2006 | - | 1 | - | - |
| 2007-2012 | 0.007206 | 0.6797 | 0.5128 | 0.9008 |
| 2013+ | 0.000936 | 0.4575 | 0.2879 | 0.7270 |
| **Patient Reported Progression in 2^nd^ Line** |  |  |  |  |
| No |  | 1 |  |  |
| Yes | 0.0203 | 1.422 | 1.056 | 1.913 |
| **Ever Had Regorafenib** |  |  |  |  |
| No |  | 1 |  |  |
| Yes | 0.0000000984 | 0.4584 | 0.3441 | 0.6107 |
| **Age at Start of Treatment** | 0.00239 | 1.014 | 1.005 | 1.024 |
| **Gender** |  |  |  |  |
| Female | - | 1 |  |  |
| Male | 0.0376 | 1.299 | 1.015 | 1.663 |
| **Patient Knows Their Mutation** |  |  |  |  |
| Has reported their mutation | - | 1 | - | - |
| Has not reported their mutation | 0.0000298 | 1.676 | 1.315 | 2.137 |
| **Mutation** |  |  |  |  |
| KIT exon 11 | - | 1 | - | - |
| KIT exon 9 | *p* = 0.36598 | 1.2178 | 0.7944 | 1.8671 |
| D842V (PDGFRA, exon 18) | *p* = 0.14164 | 0.5058 | 0.2039 | 1.2552 |
| KIT/PDGFRA WT | *p* = 0.00108 | 0.4311 | 0.2603 | 0.7138 |
| Rare KIT/PDGFRA mutations combined  (KIT exons 13,17, PDGFRA exons 12, 14) | *p* = 0.25523 | 1.5686 | 0.7223 | 3.4063 |
| Unknown mutation | *p* = 0.00822 | 1.4638 | 1.1035 | 1.9417 |

| Table S7– Multi-variable analysis – OS from start of 3L, Cox regression | | | | |
| --- | --- | --- | --- | --- |
|  | p value | Hazard Ratio | 95% CI for HR | |
|  |  |  | Lower | Upper |
| **Start Category** |  |  |  |  |
| 2002-2006 |  | 1 |  |  |
| 2007-2012 | 0.190 | 0.817 | 0.603 | 1.106 |
| 2013+ | 0.894 | 0.963 | 0.554 | 1.67 |
| **Patient Reported Progression in 2^nd^ Line** |  |  |  |  |
| Yes | <0.001 | 1.812 | 1.330 | 2.470 |
| No |  | 1 |  |  |
| **Ever Had Regorafenib** |  |  |  |  |
| No |  | 1 |  |  |
| Yes | <0.001 | 0.3891 | 0.275 | 0.550 |
| **Age at Start of Treatment** | 0.009 | 1.013 | 1.003 | 1.024 |
| **Gender** |  |  |  |  |
| Male | 0.142 | 1.214 | 0.937 | 1.572 |
| Female |  | 1 |  |  |
| **Mutation** |  |  |  |  |
| KIT exon 11 |  | 1 |  |  |
| KIT exon 9 | 0.390 | 1.209 | 0.785 | 1.862 |
| D842V (PDGFRA, exon 18) | 0.021 | 0.339 | 0.135 | 0.849 |
| KIT/PDGFRA WT | 0.004 | 0.451 | 0.263 | 0.773 |
| Rare KIT/PDGFRA mutations combined  (KIT exons 13,17, PDGFRA exons 12, 14) | 0.230 | 1.616 | 0.739 | 3.534 |
| Unknown mutation | 0.208 | 1.212 | 0.893 | 1.636 |
